# The Catalase Gene *MrCat1* Contributes to Oxidative Stress Tolerance, Microsclerotia Formation, and Virulence in the Entomopathogenic Fungus *Metarhizium rileyi*

**DOI:** 10.3390/jof10080543

**Published:** 2024-08-02

**Authors:** Yu Su, Xuyi Wang, Yuanli Luo, Huan Jiang, Guiting Tang, Huai Liu

**Affiliations:** 1 College of Plant Protection, Southwest University, Chongqing 400716, China; 15978967939@163.com; 2 Southeast Chongqing Academy of Agricultural Sciences, Chongqing 408000, China; wxyi135@163.com (X.W.); luoyuanli163@163.com (Y.L.); jhhyw521@163.com (H.J.); guitingtang616@163.com (G.T.)

**Keywords:** *Metarhizium rileyi*, catalase, oxidative stress tolerance, microsclerotium, virulence

## Abstract

Catalases play a crucial role in the metabolism of reactive oxygen species (ROS) by converting H_2_O_2_ into molecular oxygen and water. They also contribute to virulence and fungal responses to various stresses. Previously, the *MrCat1*-deletion mutant (Δ*MrCat1*) was generated using the split-marker method in *Metarhizium rileyi*. In this study, the *Cat1* gene was identified, and its function was evaluated. Under normal culture conditions, there were no significant differences in colony growth or dimorphic switching between Δ*MrCat1* and the wild-type (WT) strains. However, under oxidative stress, the colony growth was inhibited, and the yeast–hyphal transition was suppressed in the Δ*MrCat1* strain. Hyperosmotic stress did not differ significantly between the two strains. In the Δ*MrCat1* strain, microsclerotia (MS) formation was delayed, resulting in less uniform MS size and a 76% decrease in MS yield compared to the WT strain. Moreover, the Δ*MrCat1* strain exhibited diminished virulence. Gene expression analysis revealed up-regulation of Δ*MrCat1, MrCat2*, *MrCat4*, and *MrAox* in the Δ*MrCat1* strain. These findings indicate that the *MrCat1* gene in *M. rileyi* is essential for oxidative stress tolerance, MS formation, and virulence.

## 1. Introduction

*Metarhizium rileyi* is a valuable entomopathogenic fungus known for its ability to infect lepidopterous pests, particularly *Noctuidae* spp., making it a potential candidate for insect biocontrol [1,2,3,4,5]. However, the sporulation of *M. rileyi* is dependent on specific conditions such as light stimulation and a maltose carbon source, which limits its commercial potential. To overcome this limitation, researchers have successfully induced microsclerotia (MS) formation in *M. rileyi* using liquid-amended media (AM) [6]. These MS are specialized, hyperpigmented hyphal aggregations with a diameter of 50–600 μm. Compared to *M. rileyi* conidia, MS have higher production efficiency and exhibit longer persistence in the field. Other entomopathogenic fungi like *Metarhizium brunneum* [7] and *Metarhizium anisopliae* [8] also produce MS and have been used in insect biocontrol.

In filamentous fungi, studies have shown that reactive oxygen species (ROS) play a critical role in various aspects of cell physiology, cellular differentiation, cell signaling, and defense against pathogens [9]. In the case of *M. rileyi*, comparative transcriptome analysis has been conducted to investigate the molecular mechanisms underlying MS development. This analysis revealed that oxidative stress is a key factor in MS development [10]. Several genes involved in MS development have been identified in *M. rileyi*. For example, the small GTPase *RacA* and *Cdc42*, along with their regulatory factors *Cdc24* and *Bem1*, regulate MS formation by controlling ROS generation [11,12]. Additionally, the NADH:flavin oxidoreductase/NADH oxidase gene (*Nox*) and alternative oxidase (*Aox*) genes are involved in MS formation by regulating intracellular H_2_O_2_ concentrations [13,14,15]. Defects in the transmembrane sensor genes *Sho1* and *Sln1* [16], as well as the deletion of two MAPK genes, *Hog1* and *Slt2*, have also been found to inhibit MS formation [17].

Catalases are crucial enzymes in the metabolism of ROS. They, along with superoxide dismutases (SODs) and catalases, convert superoxide and H_2_O_2_ into water and molecular oxygen [18,19,20,21]. Previous studies have highlighted the important adaptive role of catalases in response to environmental stress. For example, in *Beauveria bassiana*, five catalase genes (*CatA*, *CatB*, *CatP*, *CatC,* and *CatD*) have been implicated in the regulation of virulence and tolerance to oxidative stress, high temperatures, and UV-B radiation [22]. Overexpression of the *Cat1* gene in *M. anisopliae* has been shown to enhance resistance to exogenous H_2_O_2_, reduce germination time, and increase virulence [23]. In contrast, *Neurospora crassa* exhibited defects in the survival of conidia under oxidative and light-induced stress [24], while *Cat3* mutants exhibited growth and differentiation defects [25].

In *Aspergillus oryzae*, the *CatB* gene has been implicated in the detoxification of oxidative stress [26]. In the phytopathogenic fungus *Claviceps purpurea*, catalase has been shown to suppress the host defense system [27]. Furthermore, peroxisomal catalases may be involved in insect hydrocarbon catabolism [28]. These results collectively demonstrate the importance of fungal catalase genes in detoxification, cellular differentiation, and catabolism. However, there have been no reports on the involvement of catalase genes in MS formation. In this study, we isolated genomic DNA and cDNA of the *Cat1* gene in *M. rileyi* and investigated its role in oxidative stress tolerance, MS formation, and virulence through gene deletion experiments.

## 2. Materials and Methods

### 2.1. Strains and Growth Conditions

The *M. rileyi* strain Nr01 utilized in this study was obtained from the Engineering Research Center of Fungal Insecticides located in Chongqing, China. The mutant strain Δ*MrCat1*, lacking the *MrCat1* gene, was previously generated and confirmed by a study [29]. Both the wild-type (WT) and Δ*MrCat1* strains were cultured on solid SMAY media containing 40 g L^−1^ maltose, 15 g L^−1^ yeast extract powder, and 10 g L^−1^ peptone. Blastospores were collected during the early stages of growth, while conidia were collected after spore production by suspending the colonies in a sterile 0.05% Tween-80 solution and filtering them through lens wiping paper. The resulting blastospores of the WT and Δ*MrCat1* strains were washed and suspended in sterile phosphate-buffered saline (PBS) using a process involving three rounds of centrifugation. The *M. rileyi* spores were then cultured in liquid AM medium at 28 °C with continuous shaking at 250 rpm, following a described protocol [6].

### 2.2. Cloning the MrCat1 Gene of M. rileyi

The partial *MrCat1* sequence in this study was obtained from transcriptome data reported by Song [10]. To obtain the full cDNA and genomic sequences, the utilization of the fusion primer and nested integrated PCR (FPNI-PCR) method described by Wang [30]. The amino acid sequence was deduced by performing BlastX searches in GenBank. Signal peptide prediction was carried out by signalP 6.0, a web-based tool available at http://www.cbs.dtu.dk/serbices.signal/ (accessed on 21 February 2023). The conserved domain of *MrCat1* was predicted using the SMART web resource, available at http://smart.embl.de/ (accessed on 21 February 2023). The obtained sequences were aligned using MUSCLE with default settings, and an unrooted phylogenetic tree was generated using the Maximum Likelihood method in MEGA-X (https://www.megasoftware.net/) (accessed on 25 February 2023) with a 1000 replicates bootstrap test, as detailed by Kumar [31].

### 2.3. Gene Expression Patterns of M. rileyi WT and ΔMrCat1 Strains during MS Development

The transcription levels of *MrCat1*, *MrCat2*, *MrCat4*, and *MrAox* were quantified using real-time quantitative PCR (RT-qPCR) at different time points during the development of MS. The WT and Δ*MrCat1* strains were introduced into flasks containing 100 mL of liquid AM, along with 0.5 mL of conidia suspension (1 × 10^8^ sp mL^−1^). The flasks were then incubated with agitation at 28 °C and 250 rpm for 1.5–7 days. Samples were collected at specific time intervals corresponding to different stages of development: germinating spores (1.5–2 days), yeast-like cells (2.5–3 days), MS initiation and hyphal period (3.5–4 days), MS formation (4.5–5 days), MS maturation (5.5–6 days), and secondary mycelial growth (6.5–7 days). After centrifugation and two washes with sterile distilled water, total RNA was extracted using TRIzol^®^ reagent (Invitrogen, Carlsbad, CA, USA). First-strand cDNA was then synthesized following the manufacturer’s protocol using SuperScript II Reverse Transcriptase (Invitrogen, Carlsbad, CA, USA). RT-qPCR was performed using SYBR^®^ Green II mix (TaKaRa, Shiga, Japan) according to the manufacturer’s instructions. The primers used to assess the expression levels of *MrCat1*, *MrCat2*, *MrCat4*, and *MrAox* are detailed in Table A1. Each sample was prepared in triplicate, and each reaction was carried out three times. The *MrTef* and *MrTub* genes were employed as reference genes for normalization.

### 2.4. H_2_O_2_ Sensitivity of WT and ΔMrCat1 Strains

To assess the sensitivity to H_2_O_2_ in the WT and Δ*MrCat1* strains, the filter paper method was employed. Conidial suspensions (1 × 10^6^ sp mL^−1^) of the WT and Δ*MrCat1* strains were spread onto plates. On the center of each plate, a 4-mm filter paper containing 800, 1600, or 3200 mM H_2_O_2_ was placed. For colony morphology evaluation, conidial suspensions (1 × 10^7^, 1 × 10^6^, and 1 × 10^5^ sp mL^−1^) of the WT and Δ*MrCat1* strains were pipetted onto SMAY plates and SMAY plates supplemented with 2 mM H_2_O_2_. All treatments were then incubated at 25 °C.

### 2.5. Hyperosmolarity Tolerance of WT and ΔMrCat1 Strains

To evaluate the tolerance of the WT and Δ*MrCat1* strains to NaCl, KCl, LiCl, and sorbitol, solid SMAY media was used. Three concentrations of conidial suspensions (1 × 10^7^, 1 × 10^6^, and 1 × 10^5^ sp mL^−1^) were pipetted onto plates for the evaluation of colony morphology.

### 2.6. MS Formation of WT and ΔMrCat1 Strains

To examine the MS formation capacities of the WT and Δ*MrCat1* strains, a conidial suspension (1 × 10^8^ sp mL^−1^) was inoculated into 100 mL of liquid AM. The inoculated cultures were then incubated at 28 °C with shaking at 250 rpm for 3.5–6.0 days. During the incubation period, the formation of MS yield was monitored, and the yield of MS was assessed. Additionally, the morphologies of the MS structures were observed using a microscope.

### 2.7. Virulence Assays

To determine the virulence of the WT and Δ*MrCat1* strains, third-instar larvae of *Prodenia litura* were used in the experiment. The larvae were immersed in three different concentrations of conidial suspensions: 2.5 × 10^7^ sp mL^−1^, 5 × 10^7^ sp mL^−1^, and 1 × 10^8^ sp mL^−1^. Control experiments were also conducted using a 0.05% Tween-80 solution without the fungus. After treatment, the insects were incubated at 26 °C, and mortality was recorded every 24 h. The median lethal time (LT_50_) values were calculated using the minimum squares method, and the median lethal concentration (LC_50_) values were calculated using the linearized regression model (LRM) method. Each treatment was repeated three times, with 15 larvae per replicate, to ensure reliable and statistically significant results.

### 2.8. Data Statistical Analysis

To assess variations in the relative expression levels of the genes among the samples, a variance analysis was conducted. Following the variance analysis, multiple comparisons were performed using Duncan’s least significant range (LSR) tests in the SPSS statistical software (SPSS 16.0, SPSS Inc., Armonk, NY, USA). If a column lacks a common superscript letter, it indicates a significant difference compared to other columns (*p* < 0.05). On the other hand, columns labeled with the same letter were not found to be significantly different at the 5% level, based on Duncan’s multiple range test.

## 3. Results

### 3.1. Features of MrCat1 in M. rileyi

**Figure 1:**
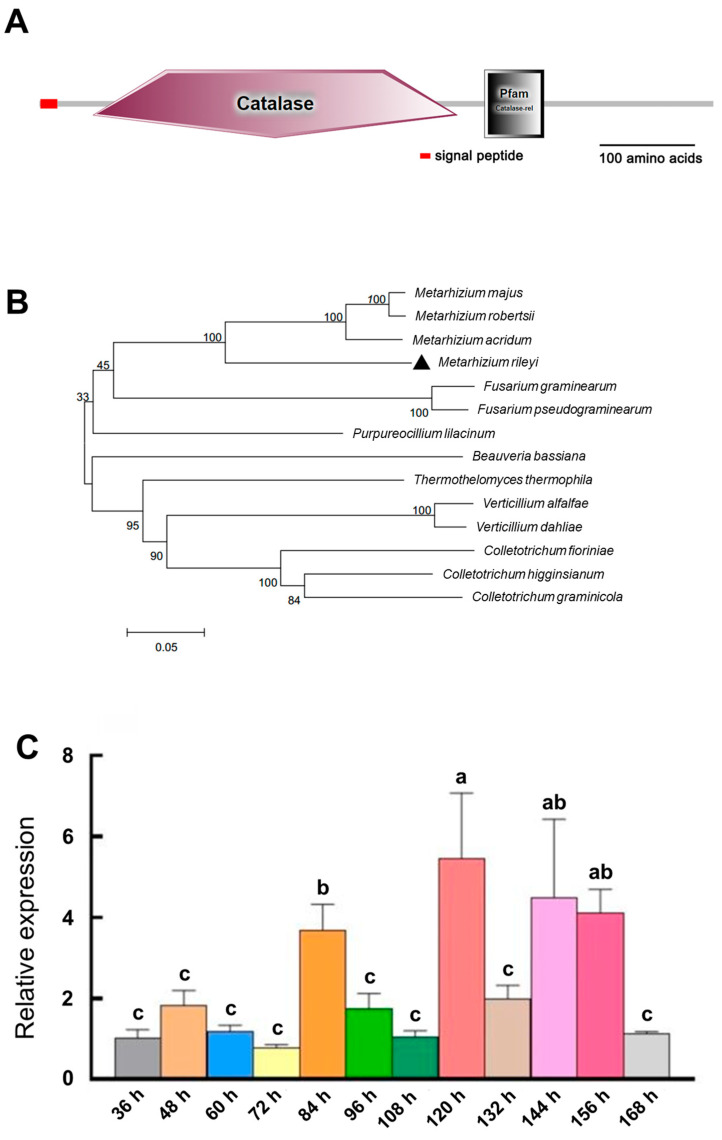
Features of *MrCat1* in *Metarhizium rileyi*. (**A**) The schematic of the protein primary structure encoded by the *MrCat1* gene, with the small red box indicating the signal peptide structure. (**B**) A phylogenetic tree inferred from *Cat1* DNA sequence alignment using the neighbor-joining (NJ). The aligned sequences of *Cat1* are from *Metarhizium rileyi* (OAA38085.1) *Metarhizium acridum* CQMa 102 (XM_007811861.1), *Metarhizium majus* ARSEF 297 (XM_014726471.1), *Purpureocillium lilacinum* (XM_018322662.1), *Fusarium graminearum* PH-1 (XM_011328072.1), *Fusarium pseudograminearum* CS3096 (XM_009262196.1), *Thermothelomyces thermophila* ATCC 42464 (XM_003662984.1), *Colletotrichum higginsianum* IMI 349063 (XM_018298034.1), *Colletotrichum fioriniae* PJ7 (XM_007598278.1), *Colletotrichum graminicola* M1.001 (XM_008092892.1), *Verticillium alfalfae* VaMs.102, (XM_003001071.1), *Verticillium dahliae* VdLs.17 (XM_009658953.1), *Metarhizium robertsii* ARSEF 23 (XM_007823877.1), and *Beauveria bassiana* (JX050139.1). (**C**) RT-qPCR analysis to assess the expression of *MrCat1*. Total RNA was isolated during different periods of MS development from the WT strain. Results are mean relative expression ± SD. The letters (a, b, c) indicated a significant difference compared to a column lacking a common superscript letter (*p* < 0.05). Conversely, columns labeled with the same letter were not significantly different at the 5% level.

The *MrCat1* gene was identified from the expressed sequence tag (EST) of the *Cat1* gene isolated from the transcriptome library of *M. rileyi*. It has a full-length sequence of 2561 base pairs (bp) and encodes 716 amino acid residues. Comparison of cDNA and genomic sequences revealed the presence of four introns in *MrCat1*. Similar to other fungal genes, it contains a signal peptide for secretion and exhibits a typical catalase structural domain (Figure 1A), indicating that it is likely an exocrine protein. The predication molecular weight of the *MrCat1* is 79.085 KDa, and its isoelectric point (pI) is 5.92. A phylogenetic analysis using MEGA-X software revealed that *MrCat1* is closely associated with *Metarhizium* spp. (Figure 1B). The deduced amino acid sequence of *MrCat1* showed similarities with catalase in *Metarhizium acridum* (78.85% identity) [32] and catalaseB in *Purpureocillium lilacinum* (69.18% identity) [33], suggesting functional similarities with these enzymes.

To assess the expression of *MrCat1* during MS development, RT-qPCR analysis was performed. The results showed that *MrCat1* was expressed at all stages of MS development. It exhibited significantly up-regulated during MS initiation (84 h, 3.6-fold), MS formation (120 h, 5.4-fold), MS maturation (144 h, 4.4-fold), and the early stage of secondary mycelial growth (156 h, 4.1-fold) compared to the germinating spore period (36 h). These findings suggest that the *MrCat1* may play a potential role in hyphal growth and MS formation (Figure 1C).

### 3.2. Deletion of MrCat1 Impaired the Tolerance to H_2_O_2_ in M. rileyi

**Figure 2:**
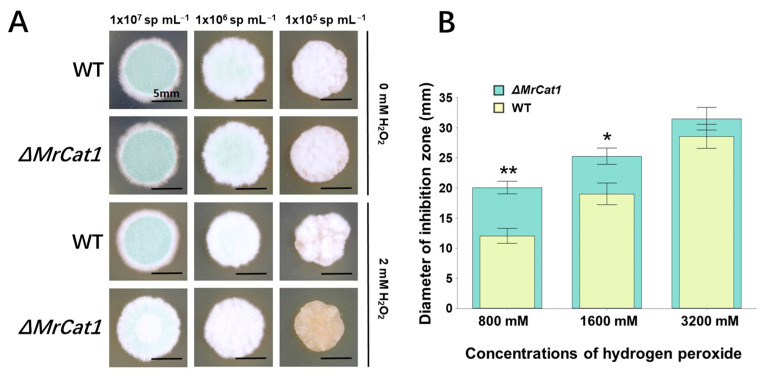
Deletion of *MrCat1* impaired the tolerance to H_2_O_2_ in *M. rileyi*. (**A**) The colony morphology of the wild-type (WT) strain and the Δ*MrCat1* mutant strain observed on SMAY plates or SMAY plates supplemented with 2 mM H_2_O_2_ for 7 days. Three concentrations of conidial suspensions (1 × 10^7^, 1 × 10^6^, and 1 × 10^5^ sp mL^−1^) were pipetted onto the plates. (**B**) The diameter of H_2_O_2_ inhibition zones measured at H_2_O_2_ concentrations of 800, 1600, and 3200 mM. Values are presented as the relative mean ± SD from five independent assays. Standard error bars indicate variation in measurements. * *p* < 0.05, ** *p* < 0.01, compared with WT grown at the same concentration of H_2_O_2_.

To investigate the role of *MrCat1* in H_2_O_2_ metabolism, a deletion mutant of *MrCat1* was created in *M. rileyi* using the split-marker method [29]. Both the WT and Δ*MrCat1* conidia were grown on SMAY plates supplemented with 2 mM H_2_O_2_ at a temperature of 25 °C for 7 days. Notably, there were no noticeable differences in morphology between the colonies of the WT and Δ*MrCat1* strains. However, a delay in the dimorphic switch was observed in the Δ*MrCat1* strain grown on SMAY plates with 2 mM H_2_O_2_. Specifically, the dimorphic switch was delayed by approximately 1 and 2 days compared to the WT strain (Figure 2A). In H_2_O_2_ inhibition assays, the Δ*MrCat1* strain exhibited larger inhibition zones compared to the WT strain. The size of the inhibition zones for the Δ*MrCat1* strain grown at H_2_O_2_ concentrations of 800, 1600, and 3200 mM were 1.7, 1.3, and 1.1 times larger, respectively, than those of the WT strain under the same conditions (Figure 2B). Variance analysis revealed that the diameter of the inhibition zones for the Δ*MrCat1* strain grown at an H_2_O_2_ concentration of 800 mM was significantly different from the WT strain (*p* < 0.01).

### 3.3. Deletion of MrCat1 Did Not Affect the Hyperosmotic Tolerance of M. rileyi 

**Figure 3:**
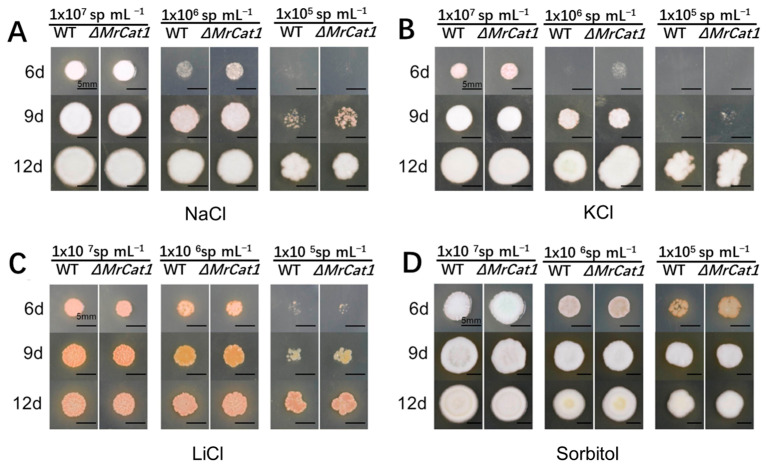
Deletion of *MrCat1* did not affect the hyperosmotic tolerance of *M. rileyi*. The colony morphology of the WT and Δ*MrCat1* strains observed on SMAY plates supplemented with (**A**) 1 M NaCl, (**B**) 1 M KCl, (**C**) 0.04 M LiCl, and (**D**) 1 M sorbitol for 6, 9, and 12 days, respectively.

Both the WT and Δ*MrCat1* conidia were cultivated on SMAY plates supplemented with different hyperosmotic conditions, including 1 M NaCl, 1 M KCl, 0.04 M LiCl, and 1 M sorbitol. The cultivation period lasted for 18 days at a temperature of 25 °C. The growth rate of both strains, WT and Δ*MrCat1*, was significantly hindered by the presence of hyperosmotic conditions, except for the presence of sorbitol. The growth of Δ*MrCat1* strain exhibited a promotion at the beginning of the inoculation period (6d and 9d) compared with the WT strain (Figure 3A–D). However, despite the differences in growth rate, there were no discernible differences in colony morphologies between the two strains (Figure 3A–D).

### 3.4. Deletion of MrCat1 Affected MS Development of M. rileyi

**Figure 4:**
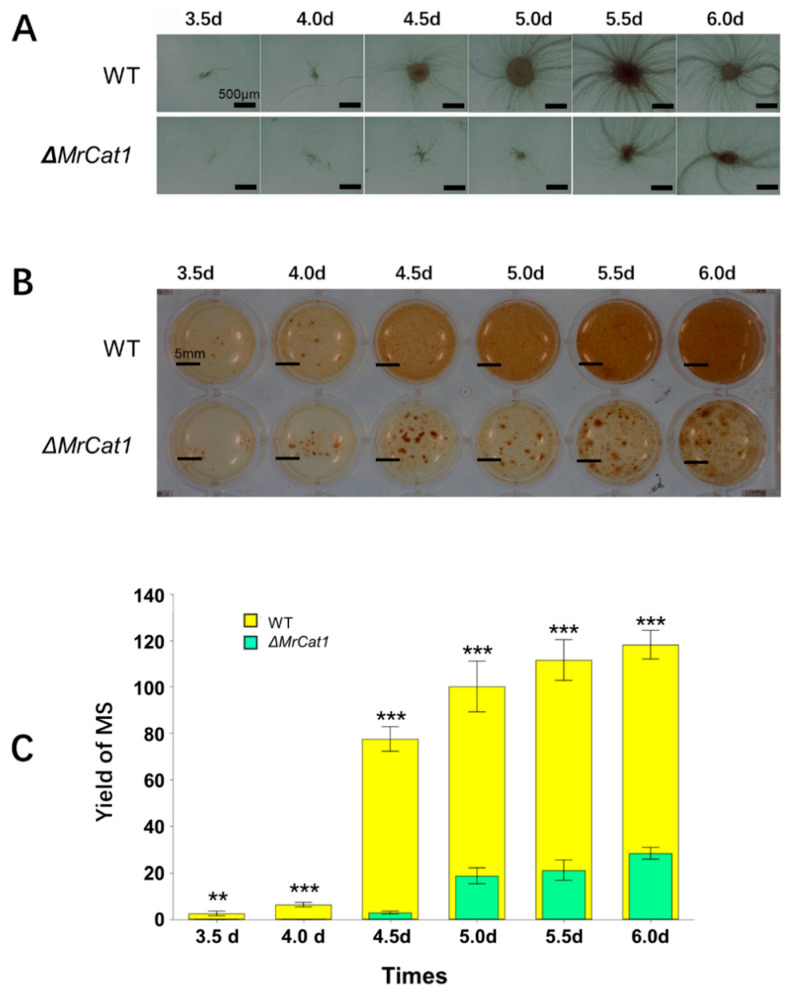
Deletion of *MrCat1* affected the MS development of *M. rileyi*. Microscopic images (**A**) and morphology (**B**) of the WT and Δ*MrCat1* strains grown in AM media from day 3.5–6. (**C**) Numbers of MS generated by the WT and Δ*MrCat1* strains from day 3.5–6. Values are presented as relative mean ± SD from three independent assays. Standard error bars indicate variation in measurements. ** *p* < 0.01, *** *p* < 0.001, compared with the WT strain at the same time point.

Both the WT and Δ*MrCat1* conidia were introduced into a liquid AM medium and subjected to agitation. The WT strains exhibited visible MS formation after a period of 3.5–4 days, while the Δ*MrCat1* strain experienced a delay in MS formation, occurring after 4.5–5 days. Furthermore, the fermentation broth of the Δ*MrCat1* strain exhibited lower viscosity and a lighter pigment compared to the WT strain (Figure 4A,B). Additionally, the size distribution of WT MS displayed greater uniformity, ranging from 70 to 300 μm, compared to the Δ*MrCat1* MS, which ranged from 50 to 1500 μm. Moreover, the MS yield of the Δ*MrCat1* strain was diminished by approximately 76% compared to the WT strain (Figure 4C).

### 3.5. Deletion of MrCat1 Resulted in Enhancing the Expression of MrCat2, MrCat4, and MrAox during MS Development of M. rileyi

**Figure 5:**
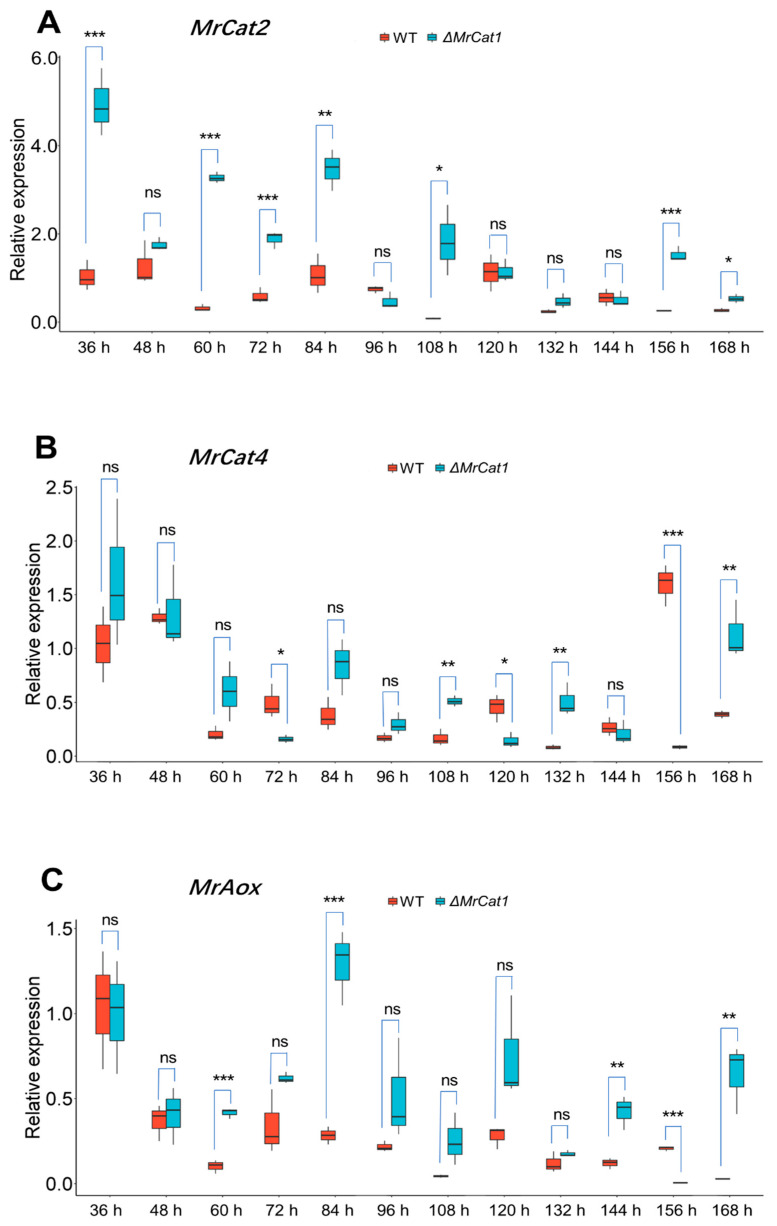
Deletion of *MrCat1* resulted in enhancing the expression of *MrCat2*, *MrCat4,* and *MrAox* during the MS formation of *M. rileyi*. RT-qPCR analysis of *MrCat2* (**A**), *MrCat4* (**B**), and *MrAox* (**C**) expression. Total RNA was isolated during MS development from the WT and Δ*MrCat1* strains. Results are mean relative expression ± SD. * *p* < 0.05, ** *p* < 0.01, *** *p* < 0.001, ‘ns’ represented no significant difference, compared between WT and Δ*MrCat1* at the same time point.

The expressions of *MrCat2*, *MrCat4*, and *MrAox* genes were analyzed during different stages of MS development, including spore germinating, MS initiation, formation stage, and secondary mycelial growth phase, in both the WT and Δ*MrCat1* strains of *M. rileyi*. Remarkably, the transcription levels of the *MrCat2* and *MrAox* were consistently higher in the Δ*MrCat1* strain compared to the WT strain (Figure 5A,C). Specifically, for *MrCat2*, the transcription levels were higher during the spore germinating to MS initiation period (36 h–84 h) and exhibited higher expression in the early period of MS formation (108 h) and early period of secondary mycelial growth (156 h). The most significant compensation occurred at 60 h, where *MrCat2* was up-regulated by 10.2-fold (Figure 5A). 

Similarly to *MrAox*, the transcription levels were higher from the yeast-like cell period to MS maturation (60 h–144 h) and were up-regulated in the later period of secondary mycelial growth (168 h). The most significant compensation occurred at 168 h, where *MrAox* was up-regulated by 22.1-fold. However, transcription levels of the *MrCat4* gene were prominent during the secondary mycelial growth phase and exhibited irregular compensation between the WT and Δ*MrCat1* strains. Taken together, the results indicate that *MrCat2*, *MrCat4,* and *MrAox* genes exhibit significantly higher expression in the Δ*MrCat1* strain compared to the WT strain during the MS initiation period (84 h), which is a key time point for MS initiation (Figure 5A–C). This suggests that in *M. rileyi*, *MrCat1*, *MrCat2,* and *MrAox* may have shared functions during MS formation.

### 3.6. Deletion of MrCat1 Reduced the Virulence of M. rileyi 

**Figure 6:**
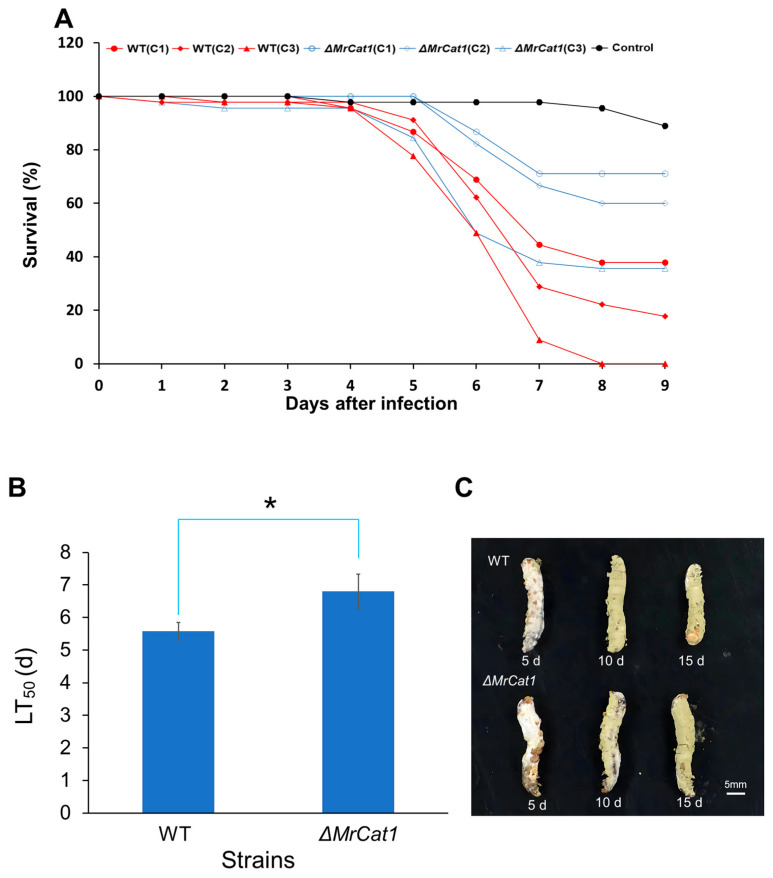
Deletion of *MrCat1* reduced the virulence of *M. rileyi*. (**A**) The survival rates of *P. litura* larvae infected by the WT and Δ*MrCat1* strains measured at different concentrations of spore suspensions (C1: 2.5 × 10^7^ sp mL^−1^, C2: 5.0 × 10^7^ sp mL^−1^, C3: 1.0 × 10^8^ sp mL^−1^). (**B**) LT_50_ of the WT and Δ*MrCat1* strains against *P. litura* larvae under spore concentration of 1 × 10^8^ sp mL^−1^. * *p* < 0.05, compared between WT and Δ*MrCat1*. (**C**) The morphology of muscardine cadavers infected by WT and Δ*MrCat1*. The *P. litura* larvae were killed by WT and Δ*MrCat1* for 5, 10, and 15 days.

To examine the effects of *MrCat1* deletion on the pathogenicity of *M. rileyi*, insect bioassays were conducted to infect *P. litura* larvae. The survival rates and LT_50_ were determined and compared between the WT and Δ*MrCat1* strains. After infection, it was observed that the survival rates of *P. litura* larvae infected by the Δ*MrCat1* strain were significantly higher than those infected by the WT strain at the same concentration (2.5 × 10^7^ sp mL^−1^, 5.0 × 10^7^ sp mL^−1^, 1.0 × 10^8^ sp mL^−1^) (Figure 6A). Moreover, the mortality of the Δ*MrCat1* stain at the concentration of 2.5 × 10^7^ sp mL^−1^ and 5.0 × 10^7^ sp mL^−1^ was less than 50% at the time point of 9 d. However, at the concentration of 1.0 × 10^8^ sp mL^−1^, the LT_50_ was 6.81 ± 0.52 days. In comparison, the LT_50_ for the WT at the concentrations of 2.5 × 10^7^ sp mL^−1^, 5.0 × 10^7^ sp mL^−1^, and 1.0 × 10^8^ sp mL^−1^ were 7.29 ± 0.57 days, 6.56 ± 0.33 days, and 5.57 ± 0.27 days, respectively. The LT_50_ of the Δ*MrCat1* strain was prolonged 1.2 times compared with the WT strain at a concentration of 1.0 × 10^8^ sp mL^−1^, which exhibited a significant difference (*p* < 0.05; Figure 6B). The LC_50_ of Δ*MrCat1* and WT at the time point of 8 d were 3.077 × 10^7^ sp mL^−1^ and 9.831 × 10^6^ sp mL^−1^, respectively. However, there was no noticeable difference in the morphology of muscardine cadavers between the WT and the Δ*MrCat1* strains. Furthermore, the insect cadavers were fully covered by fungal spores or mycelia from both the WT and Δ*MrCat1* strains after insect death (Figure 6C). For more detailed information about the bioassay, please refer to Table A2.

## 4. Discussion

In this study, a catalase gene (*MrCat1*) was identified and isolated from *M. rileyi*. This gene showed up-regulation during MS formation. Sequence analysis revealed that *MrCat1* shares sequence similarity with monofunctional catalases found in other entomopathogenic fungi such as *M. anisopliae*, *B. bassiana*, and *Magnaporte grisea*. Previous studies have demonstrated the involvement of catalases in virulence and stress response. In these fungi, catalases are also known to play a role in combating oxidative stress, heat stress, hyperosmotic stress, and UV-B radiation in *B. bassiana*, *Cryptococcus neoformans*, and *M. grisea* [22,34,35]. 

In this study, no significant differences were observed in colony growth or dimorphic switching between yeast and hyphae on solid media when comparing the Δ*MrCat1* mutants and the WT strain. However, under oxidative stress conditions, both colony growth and dimorphic switching were impaired in the Δ*MrCat1* mutants compared to the WT strain. Additionally, the tolerance of conidia to H_2_O_2_ showed notable variation. These findings align with the documented role of *Cat1* in reactive oxygen species (ROS) metabolism in *B. bassiana* [22], *M. anisopliae* [23], and *M. grisea* [35]. Deletion of the *MrCat1* analog *MgCatB* in *M. grisea* resulted in accelerated hyphal growth but also caused paler pigmentation, reduced biomass, fragile conidia and appressoria, poor sporulation, and impaired melanization [35]. However, in *B. bassiana* and *M. rileyi*, the deletion of *MrCat1* did not lead to these phenotypic changes [22]. The studies have suggested that *MrCat1* in *M. rileyi* is involved in oxidative stress response and plays a role in colony growth, dimorphic switching, and conidial tolerance to H_2_O_2_. Understanding the specific mechanisms of *MrCat1* and their interactions with microsclerotia (MS) is crucial for future research in this field.

The present study also found that *MrCat1* in *M. rileyi* is significantly up-regulated during the development of mycelial sclerotia (MS) and the early stage of secondary mycelial growth. Deleting *MrCat1* resulted in a 76% reduction in MS production compared to the WT strain, as well as irregular MS morphology. These findings indicate that *Cat1* is involved in both reactive oxygen species (ROS) metabolism and MS formation in *M. rileyi*. Previous studies have established that oxidative stress plays a role in inducing sclerotium differentiation [36,37,38,39], with optimal concentrations of H_2_O_2_ promoting MS formation while high concentrations inhibiting it [13]. In the WT *M. rileyi*, gene expression analysis of *MrCat1*, *MrCat2*, and *MrCat4* during the development of MS revealed that these three catalase genes are up-regulated during sclerotial initiation and MS formation, indicating higher H_2_O_2_ concentrations during this process. The Δ*MrCat1* mutants displayed deficiencies in both MS proliferation and morphology. 

The expression levels of Δ*MrCat1* and *MrCat2* mutants were found to be higher from the spore germinating period to MS initiation (36 to 84 h) compared to the WT strain. This indicates that *MrCat2* is up-regulated as a compensatory mechanism in response to the deletion of *MrCat1* during the early stages of MS formation. This observation is similar to what has been reported in *B. bassiana*, where the disruption of one of the five catalases led to the up-regulation of one or more other catalase genes. This suggests that some of the five catalases were functionally complementary, leading to complicated phenotypic changes [22], which is consistent with the results of the present study. However, the compensatory expression pattern of *MrCat4* in the Δ*MrCat1* mutants is irregular due to the absence of *MrCat1*. In contrast, in *M. grisea*, mutants lacking *MgCatB* do not show overexpression of other catalase or catalase-related antioxidant genes [35]. These findings suggest that the function of *MrCat1* is analogous to that of *BbCatB*, but the compensatory mechanisms may differ between different fungal species.

Interestingly, in the Δ*MrCat1* mutants, it was observed that another gene involved in ROS metabolism, *MrAox*, exhibited over-expression during the yeast-like cell period to MS maturation (60 h–144 h). Previous studies have shown that *Aox* is involved in the metabolism of ROS generated through energy production and metabolism in fungi [40,41]. In *M. rileyi*, defects in *MrAox* have been shown to impair ROS metabolism and MS formation [14]. These findings indicate that reactive oxygen species (ROS) accumulate during microsclerotia (MS) formation. It has been established that catalases are important enzymes for scavenging ROS, as they can convert H_2_O_2_ to water and molecular oxygen. In *Beauveria bassiana*, the five catalases play distinct roles in regulating tolerance to oxidation. In the current study, it was found that *MrAox* was up-regulated to compensate for the deletion of *MrCat1*. This suggests that *Aox* may function similarly to catalases by regulating ROS metabolism during MS formation. Catalase genes have been identified as essential virulence factors in entomogenous fungi [22,23] and phytopathogenic fungi [35]. In line with previous research, the deletion of *MrCat1* in this study led to a notable decrease in virulence. However, further comprehensive studies are necessary to elucidate the underlying mechanism. Additionally, it is important to conduct further investigations to determine the potential involvement of *MrCat2* or *MrCat4* in virulence and MS formation.

## 5. Conclusions

The deletion of the *MrCat1* gene had an impact on ROS metabolism and MS development in *M. rileyi*. *MrCat2* and *MrCat4* were up-regulated as compensatory mechanisms in the absence of *MrCat1*. Additionally, the up-regulation of *MrAox* in response to *MrCat1* deletion, which has not been previously reported, suggests its involvement in compensating for the loss of *MrCat1* in *M. rileyi*.

## Data Availability

The original contributions presented in the study are included in the article; further inquiries can be directed to the corresponding author.

## Appendix A

**Table A1:** Primers used for RT-qPCR in this work.

Primers	Sequence 5′-3′
RT-tub-F	GGCAAGGTCGCTATGAAG
RT-tub-R	CTGGATGGAGGTAGAGTTAC
RT-tef-F	GTCATCGTCCTCAACCATC
RT-tef-R	CAGTCTCAACAGCCTTACC
RT-Cat1-F	TTCGTCAACGAAGAGGGTGA
RT-Cat1-R	GTGGAAGTCGGAGTTCTTGC
RT-Cat2-F	ACTGGAAGCTCAACAACCCT
RT-Cat2-R	GCGCATCTGGACATTCTTTA
RT-Cat4-F	GGCGTCCCTGATATTCCTGA
RT-Cat4-R	GGCGATGATATCCGTGTGTG
RT-Aox-F	AATCCCACTACATCCGTGGT
RT-Aox-R	GCTCTCGAGAAAGACGAACC

**Table A2:** LT_50_s of the wild-type and Δ*MrCat1* strains against *P. litura* larvae.

Strains	Concentrations of Conidial Suspensions	LT_50_ (d)
WT	2.5 × 10^7^ sp mL^−1^	7.29 ± 0.57
	5.0 × 10^7^ sp mL^−1^	6.56 ± 0.33
	1.0 × 10^8^ sp mL^−1^	5.57 ± 0.27
Δ*MrCat1*	2.5 × 10^7^ sp mL^−1^	>9 *
	5.0 × 10^7^ sp mL^−1^	>9 *
	1.0 × 10^8^ sp mL^−1^	6.81 ± 0.52

* Mortality less than 50% at the time point of 9 d, accurate LT_50_ could not be determined in this case.
